# *Ruta graveolens* L. Induces Death of Glioblastoma Cells and Neural Progenitors, but Not of Neurons, via ERK 1/2 and AKT Activation

**DOI:** 10.1371/journal.pone.0118864

**Published:** 2015-03-18

**Authors:** Maria Teresa Gentile, Claudia Ciniglia, Mafalda G. Reccia, Floriana Volpicelli, Monica Gatti, Stefano Thellung, Tullio Florio, Mariarosa A. B. Melone, Luca Colucci-D’Amato

**Affiliations:** 1 Laboratory of Molecular and Cellular Pathology, Department of Environmental, Biological and Pharmaceutical Science and Technologies, Second University of Naples, Caserta, Italy; 2 Department of Pharmacy, University of Naples “Federico II”, Naples, Italy; 3 Institute of Genetics and Biophysics “Adriano Buzzati Traverso”, CNR, Naples, Italy; 4 Section of Pharmacology, Department of Internal Medicine, University of Genova, Genova, Italy, and Centro di Eccellenza per la Ricerca Biomedica (CEBR) University of Genova, Genova, Italy; 5 Division of Neurology, Department of Clinical and Experimental Medicine and Surgery, Second University of Naples, Naples, Italy; 6 Centro Interuniversitario per la Ricerca in Neuroscienze (CIRN), Second University of Naples, Naples, Italy; Federico II University of Naples, Italy, ITALY

## Abstract

Glioblastoma multiforme is a highly aggressive brain tumor whose prognosis is very poor. Due to early invasion of brain parenchyma, its complete surgical removal is nearly impossible, and even after aggressive combined treatment (association of surgery and chemo- and radio-therapy) five-year survival is only about 10%. Natural products are sources of novel compounds endowed with therapeutic properties in many human diseases, including cancer. Here, we report that the water extract of *Ruta graveolens* L., commonly known as rue, induces death in different glioblastoma cell lines (U87MG, C6 and U138) widely used to test novel drugs in preclinical studies. *Ruta graveolens*’ effect was mediated by ERK1/2 and AKT activation, and the inhibition of these pathways, via PD98058 and wortmannin, reverted its antiproliferative activity. Rue extract also affects survival of neural precursor cells (A1) obtained from embryonic mouse CNS. As in the case of glioma cells, rue stimulates the activation of ERK1/2 and AKT in A1 cells, whereas their blockade by pharmacological inhibitors prevents cell death. Interestingly, upon induction of differentiation and cell cycle exit, A1 cells become resistant to rue’s noxious effects but not to those of temozolomide and cisplatin, two alkylating agents widely used in glioblastoma therapy. Finally, rutin, a major component of the *Ruta graveolens* water extract, failed to cause cell death, suggesting that rutin by itself is not responsible for the observed effects. In conclusion, we report that rue extracts induce glioma cell death, discriminating between proliferating/undifferentiated and non-proliferating/differentiated neurons. Thus, it can be a promising tool to isolate novel drugs and also to discover targets for therapeutic intervention.

## Introduction

Gliomas comprise several types of primary brain tumors accounting for approximately 50% of all neoplasms of the central nervous system (CNS) [1–3]. In particular, glioblastoma, IV grade glioma, is characterized by marked cell proliferation and heterogeneity, invasiveness and neoangiogenesis, presenting rapid progression and high frequency of recurrence [4, 5]. Therefore, the prognosis for the patients is extremely poor, with mean survival of about 14 months, even after the introduction of temozolomide [6, 7], currently the gold standard cytotoxic drug for gliomas, and few patients survive beyond five years [8]. Other treatment options are limited, and in most cases ineffective and the survival rate for these patients remains extremely low [9–13]. The cell type that gives origin to glioblastoma is still an open issue. It has been reported that either dysregulated neural stem cells, or dedifferentiated glial and neuronal cells are involved in tumor development [14, 15]. Besides the derivation of the tumoral cells, recent evidence suggests that the malignant features of glioblastoma, including radio-chemo-resistance, relay on a subset of tumoral cells endowed with stem-like properties. Thus, this subpopulation has been named as cancer stem-like cells, tumor initiating cells, or cancer propagating cells [16–19].

A number of molecular abnormalities have been involved in the pathogenesis of glioblastoma, including growth factors (i.e. EGF, PDGF, HGF, VEGF) and growth factors receptors (EGFR and HGFR) that are often upregulated, overexpressed and/or constitutively activated. Among the intracellular signaling cascades, Ras-ERK1/2, PI3K/AKT, p53 and Rb play a key role in promoting cellular transformation. In particular, upon alterations of tyrosine kinase receptors, ERK1/2 and PI3K/AKT constitutive signaling seem to be constantly present in glioblastoma, and combined activation of RAS and AKT in neural progenitors is sufficient to induce glioblastoma in mice [20–30]. Targeting specific molecular alterations is a strategy for the development of cancer therapy. Thus, a number of selective inhibitors of molecules and/or pathways involved in the pathogenesis of glioblastoma have been developed and some of them entered clinical trials. Nevertheless, for reasons largely unclear, clinical response is poor. Therefore, there is still an urgent need for novel and effective therapies for treating these tumors.

On this issue, natural product-based molecules represent interesting therapeutic alternatives. Over the past decades, cell culture and animal studies allowed the identification of numerous dietary and botanical natural compounds with anti-cancer effects, including curcumin, epigallocatechin gallate, ellagic acid and resveratrol, extracted from the *Curcuma longa*, from the grape skin, from the green tea and from many fruits and vegetables, respectively [31–40]. Diet or botanical-derived compounds are also considered for cancer preventive properties, due to the fact that they are less toxic, easily available, cost-effective and better accepted by patients. Recently, several clinical trials have been started to investigate the preventive and therapeutic efficacy of natural compounds.

*Ruta graveolens* L. (*R*. *graveolens*) is a perennial plant, native of the Mediterranean region but cultivated throughout Europe and many Asian countries, including China, India and Japan. *R*. *graveolens*, commonly known as rue, is known as medicinal plant since ancient times and currently used, particularly in Asian countries, for treatment of various disorders such as aching pain, eye problems, rheumatism and dermatitis [41, 42]. The extract of the plant contains more than 120 compounds of different classes of natural products such as acridone alkaloids, coumarins, essential oils, flavonoids and furoquinolines [43]. The components of *R*. *graveolens* species are of great interest in medicinal chemistry, as these compounds show a broad range of biological activities, and a number of them are already used in medicine. Alcoholic extracts of *R*. *graveolens* have been tested for anti-proliferative effect on different types of cancer cells, pointing towards a potential therapeutic effect in oncology [44–49].

The present study was aimed to assess the effects of the aqueous extract of *R*. *graveolens* on the proliferation of human glioma cells and of neural progenitors from mouse CNS, in comparison to differentiated, non-proliferating neural cells. Moreover, we evaluated the effects of two drugs, temozolomide and cisplatin, widely used in the GBM chemotherapy on proliferating and non proliferating neural cells as comparators of the *R*. *graveolens* extract. Finally, we investigated the modulation of ERK1/2 and AKT activities as molecular correlate of the biological effects of *R*. *graveolens* extract.

## Materials and Methods

### Extract Preparation

*R*. *graveolens* is not a protected species, leaves were collected from plants conserved at the Experimental Section of Medicinal Plants at the Botanical Garden of Naples, Italy with the permission of the “Orto Botanico” director Prof. Paolo De Luca. Whole leaves were harvested before the flowering stages, during spring 2013. 250 g of leaves were chopped, boiled in 1 L of distilled water at 110°C for 5 minutes. The extract was subsequently filtered through 0.22 μm filters (MILLEXGP, MILLIPORE, Bedford, MA), frozen under liquid nitrogen and lyophilized (VirTis-SP Scientific). When necessary for the experiments, the aqueous extract (a.e.) was diluted with MEM/F12 medium to standard concentration of 50 mg/ml.

### Cell cultures

A1 mes c-myc cells (A1) are an established cell line, generated in our laboratory as previously reported [50, 51]. A1 cells were cultured in MEM/F12 medium (Invitrogen, Milan, Italy) supplemented with 10% FBS (Invitrogen) and differentiated by serum withdrawal and stimulation with 1 mM cAMP (Sigma-Aldrich, Milan, Italy) and N2 supplement (Invitrogen). U87MG and U138 human glioblastoma cells (American Type Culture Collection, Rockville, MS, USA) [52–53] were cultured in DMEM (Invitrogen) supplemented with 10% FBS. C6 rat glioma cells [54] were cultured in Ham’s-F12 (Invitrogen) supplemented with 10% FBS. We investigated *R*. *graveolens* a.e. effects at the concentration of 0.01, 0.1, 1 and 10 mg/ml for 24, 48 and 72 hours. The results showed that the viability of U87MG, U138, C6 and A1 cells significantly decreased as the dose and the time increase with similar values at 1 and 10 mg/ml (data not shown). Rutin was purchased from Sigma-Aldrich and added at the indicated concentrations. For the inhibition tests, cells were pre-treated with 10 μM PD98058 (Sigma-Aldrich) or 1μM wortmannin (Sigma-Aldrich) 1h before addition of *R*. *graveolens* a.e.

### Cell proliferation assay

For *in vitro* cell proliferation assay, MTT and Trypan blue exclusion tests were performed. MTT assay: cells were seeded at 3x10^5^ cells/well in a 24 well plate in the presence or absence of 1mg/ml of *R graveolens* a.e., and cell proliferation assessed after 24, 48 and 72 hours. According to manufacturer’s recommendations, 50 μl of 3-(4,5 dymethylthiazol-2-il)-2,5 dyphenyl-2H-tetrazolium bromide (MTT) reagent (5mg/ml in PBS) was added to each well and, then, the cells were incubated at 37°C for three hours. One volume (500μl) of Stop mix solution (20% SDS in 50% dimethyl formamide) was added to each well and incubated at room temperature for a minimum of 1h. The plate was read at 550nm and at 630 nm as the reference wavelength. Same volume of medium without cells was used as blank. Results are expressed as OD.

Trypan blue exclusion test: cells were seeded at 3x10^5^ cells/well in a 24 well plate in presence or not of 1mg/ml of *R*. *graveolens* a.e. Cell proliferation was assessed 24, 48 and 72 h after *R*. *graveolens* a.e. addition. Cells were harvested and resuspended in 1ml of PBS. 0.2 ml of cell suspension were added to 0.5 ml of PBS and 0.3 ml of 0.4% of Trypan blue solution (Lonza, Walkersville, MD, USA). After 5 min at room temperature, cells were counted in a Burker’s chamber.

### Necrosis/apoptosis analysis

The occurrence of necrosis and/or apoptosis was analyzed by Tali image-based cytometer (Invitrogen). Briefly, cells were seeded at 10^6^ cells/plate in 60mm plates, treated or not with 1mg/ml of *R*. *graveolens* a.e. for 24, 48 and 72 h. Cells were harvested and resuspended in ABB (annexin binding buffer), 5 μl of Annexin V 488 Alexa Fluor was added to each sample and incubated at room temperature, in the dark, for 20 min. Cells were centrifuged and resuspended in 100 μl of ABB, 1 μl of Tali. Propidium iodide was added to each sample and incubated 5 min at room temperature, in the dark. Emitted fluorescence was analyzed by Tali image-base cytometry and expressed as number of cells/100. To confirm the data obtained by Tali image-based cytometry, a morphological apoptosis assay was performed. In particular, 48 hr after *R*. *graveolens* a.e. addition, cells were washed three times in PBS, and then fixed with 4% paraformaldehyde for 10min at room temperature (RT). Cells were rinsed with PBS, and 0.1μg/ml DAPI (4′6 diamidi-no-2-phenylindole, for nuclei staining) was added for 10 min at RT. After washing with PBS, the cells were detected with fluorescence microscopy (AxioImager M2, Zeiss, Germany), and cells with condensed and/or fragmented chromatin indicative of apoptosis were not counted as living cells. 250 fields/well were counted using a Scion Image version 4.5 software.

Caspase 3 activation was assessed by means of CPP32 Colorimetric Kit (BioVision Inc., CA, USA). Briefly, cells were treated with 1mg/ml *R*. *graveolens* a.e. for 24 and 48 hours and harvested. 1x10^6^ cells were incubated with chilled Cell Lysis Buffer for 10 minutes. Protein concentration was determined by Bradford method. 100 μg of total protein were additioned with 5μl of 4 mM DEVD-pNA caspase 3 substrate, and incubated at 37°C for 2 hours. Samples were read at 400 nm. Fold-increase in CPP32 activity was determined by comparing samples absorbance at 400 nm to the absorbance of the control extracts.

### Protein analysis

Isolation of cytosolic protein was performed by scraping the cells in lysis buffer (25 mM Tris-HCl, pH 7.4, 1 mM EDTA, 1 mM EGTA, 0.1 mM NaF, 0.1 mM Na_3_VO_4_). Protein concentration was determined by the Bradford method. 20 μg of protein extract were subjected to a 10% SDS-PAGE and electroblotted on nitrocellulose membranes, which were blocked at room temperature for 1 h in blocking solution (5% milk in TBS-T), incubated overnight at 4°C with anti-phsphoERK1/2 or anti-phosphoAKT antibodies (1 μg/ml; Millipore, Milan, Italy). Membrane was incubated with peroxidase-conjugated anti-rabbit antibodies (1:10,000; GE Healthcare Life Sciences, Milan, Italy). Proteins were revealed by ECL (Millipore). Normalization was performed with anti-ERK1/2 or anti AKT antibodies (1 μg/ml in TBS-T; Millipore) for 1 h at room temperature and incubated with peroxidase-conjugated anti-mouse IgG (1:10,000 in TBS-T). Proteins were revealed as above. The intensity of the bands was quantified by scanning densitometry using Scion Image version 4.5 software.

### Statistical analysis

In all the experiments statistical significance was determined using the two-tailed *t*-test. All the experiments were repeated at least three times. P<0.05 was considered a statistically significant difference.

## Results

### *R*. *graveolens* aqueous extract induces cell death of glioma cell lines and of proliferating mesencephalic cells.

**Fig 1:**
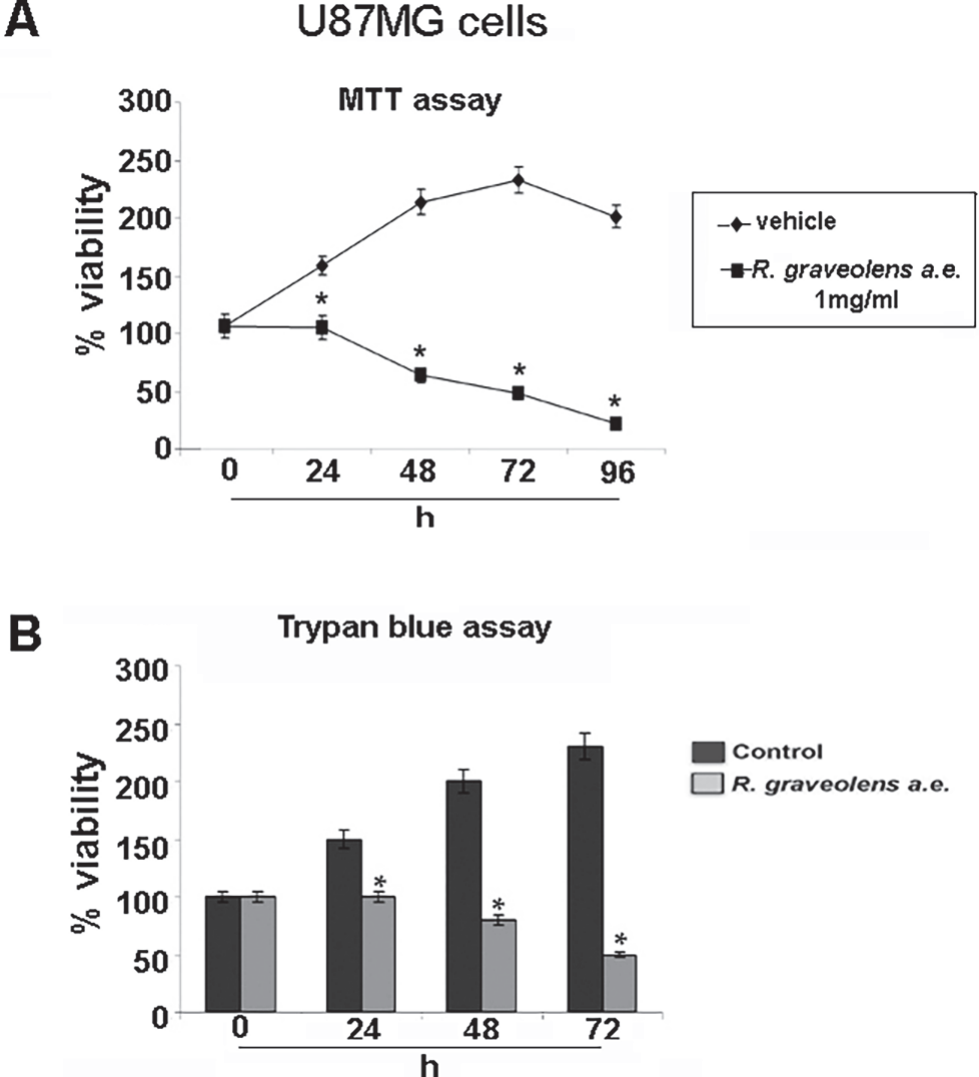
*R*. *graveolens* extract induces cell death of U87MG human glioma cells. (A) MTT assay on proliferating U87MG human glioma cells treated with vehicle (♦) or with 1mg/ml *R*. *graveolens* a.e. (■) for 24, 48, 72, 96h, *p<0,01 vs control conditions. (B) Trypan blue exclusion test on U87MG glioma cells treated (light gray) or not (dark gray) with 1mg/ml *R*. *graveolens* a.e. for 24, 48 and 72h; *<0,01 *vs* control conditions.

To assess the antiproliferative effects of *R*. *graveolens* water extract, MTT reduction assay and Trypan blue exclusion test were performed on the human glioblastoma cell line U87MG. In particular, incubation for 24 h with 1 mg/ml *R*. *graveolens* a.e determined cell proliferation arrest, as compared to vehicle treated cells, and induced cell death for treatments of 48, 72 and 96 h (Fig. 1A). These results were confirmed by Trypan blue exclusion test (Fig. 1B). Similar results were also obtained in U138 and C6 human and rat glioma cells, respectively (S1 Fig). Moreover, as expected, MTT reduction assay, performed on the same cell lines in the presence of temozolomide (TMZ) or cisplatin (CIS), two alkylating agents currently used to treat human GBM, showed that the two drugs induced a significant cell death already after 24 h of treatment, (data not shown).

**Fig 2:**
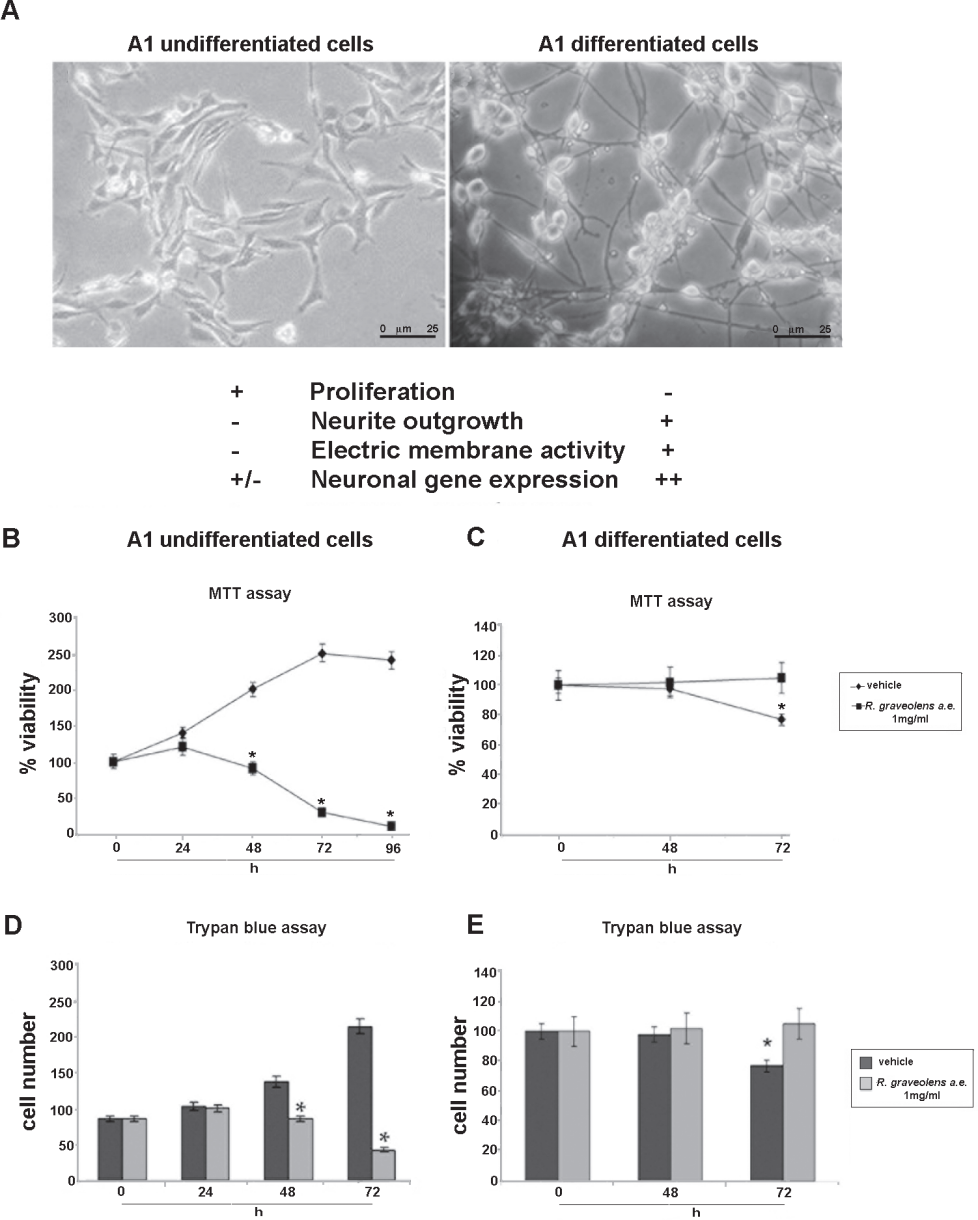
*R*. *graveolens* a.e. induces cell death in proliferating but not in differentiated A1 cells. (A) Microphotographs of the mouse mesencephalic embryonic cell line A1 mes c-myc (A1). They are proliferating/undifferentiated in the presence of serum (left panel) but acquire a neuronal phenotype upon serum withdrawal and stimulation with cAMP (right panel). (B) MTT assay on proliferating A1 cells in control conditions (♦) or treated with 1mg/ml *R*. *graveolens* a.e. (■) for 24, 48, 72 and 96 hours. *p<0.01 *vs* controls. (C) MTT assay on differentiated A1 cells in control conditions (♦) or treated with 1mg/ml *R*. *graveolens* a.e. (■) for 48 and 72 hours *p<0.01 *vs* controls. (D) Trypan blue exclusion test on proliferating A1 cells treated (light grey) or not (dark grey) with 1mg/ml *R*. *graveolens* a.e. for 24, 48 and 72h; *p<0.01 *vs* controls. (E) Trypan blue exclusion test on differentiated A1 cells treated (light gray) or not (dark gray) with 1mg/ml *R*. *graveolens* a.e. for 48 and 72h; *p<0.01 *vs* controls.

**Fig 3:**
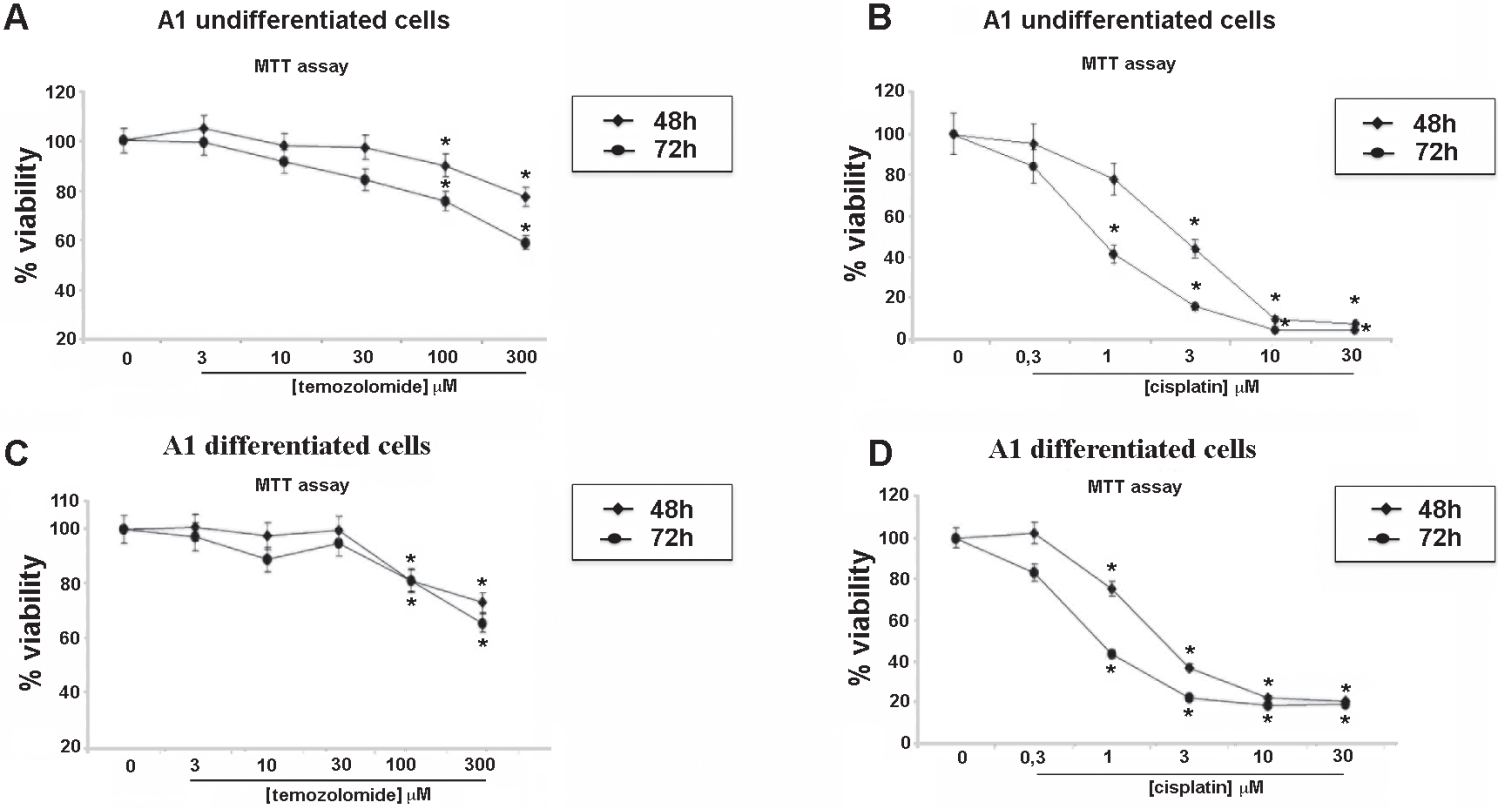
Temozolomide and cisplatin affect viability of proliferating and differentiated A1. (A-B) MTT assay on proliferating A1 cells treated with increasing concentrations of temozolomide (A) and cisplatin (B) for 48 (♦) and 72 (■) hours; *p<0.01 *vs* controls; (C-D) MTT assay on differentiated A1 cells treated with increasing concentrations of temozolomide (C) and cisplatin (D) for 48 (♦) and 72 (■) hours; *p<0.01 *vs* controls.

Similar experiments were performed on A1 cells. A1 cells derived from E11 mouse mesencephalon were immortalized by means of infection with a c-myc-carrying retroviral vector. These cells proliferate and remain undifferentiated when grown in the presence of serum whereas they cease to proliferate and differentiate, ensuing neurite outgrowth, neuronal electrophysiological properties, and expression of neuronal markers, when serum is withdrawn and cAMP is added [50] (Fig. 2A). MTT reduction assay and Trypan blue exclusion test were performed on proliferating-undifferentiated and differentiated-non proliferating A1 cells to evaluate the effects of *R*. *graveolens* a.e on cell viability. As shown in Fig. 2B, the treatment of A1 cells with 1 mg/ml *R*. *graveolens* a.e. was able to inhibit cell proliferation in proliferating A1 cells after 24h, and to induce cell death after 48 (viability decreases of about 120% as compared to vehicle treated conditions) and 72h (viability decreases of about 200% as compared to vehicle treated conditions). Trypan blue exclusion test confirmed these results showing that 1mg/ml *R*. *graveolens* a.e. significantly decreased the number of viable proliferating A1 cells, after 48 and 72 h of treatment (Fig. 2D). Conversely, both MTT assay and Trypan blue viability test performed on differentiated, non-proliferating A1 cells, revealed no statistically significant difference between control and treated cells after 48 hours from the addition of 1 mg/ml *R*. *graveolens* a.e (Fig. 2C, E). Moreover, MTT reduction assay was performed on A1 cells, in both undifferentiated and differentiated conditions, treated with TMZ and CIS. CIS and TMZ caused a significant cytotoxicity in both proliferating (Fig. 3A and B) and differentiated-non proliferating A1 cells (Fig. 3C and D).

### *R*. *graveolens* aqueous extract induces activation of ERK1/2 and AKT pathways.

**Fig 4:**
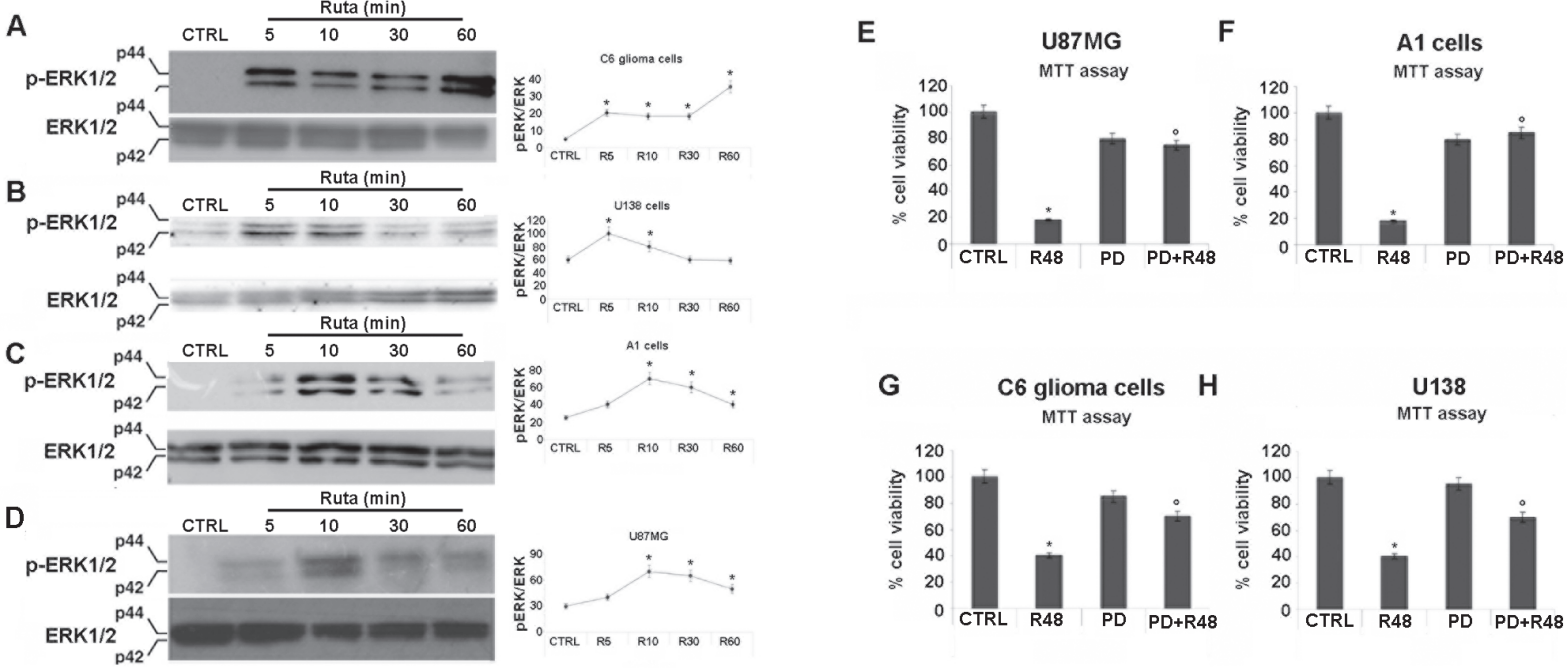
*R*. *graveolens* a.e. is able to induce ERK1/2 phosphorylation in glioma and in A1 proliferating neural cells. Western blotting detection of p-ERK1/2 and ERK1/2 proteins in C6 glioma cells (A), in U138 glioma cells (B), in A1 cells (C) and in U87MG (D) treated with 1mg/ml *R*. *graveolens* a.e. for 5, 10, 30 and 60 minutes. Two specific bands are observed respectively at 44 and 42 kDa. Each blot is representative of three separate experiments. The graphs show the relative quantitation of p-ERK1/2 and ERK1/2 in the different cell lines. Data are expressed as ratios of p-ERK/ERK. Asterisks represent p<0.05 *vs* controls. (G-H) MTT assay in U87MG cells (E), A1 cells (F), C6 glioma cells (G) and U138 cells (H) treated for 48 hours with 1mg/ml *R*. *graveolens* a.e. (R48), 10μM PD98059 (PD) or in combination (PD+R48); *p<0.01 *vs* control conditions.°p<0.05 *vs* R48.

To investigate the molecular mechanisms by which *R*. *graveolens* a.e. promotes cell death in glioma and A1 cells, we analyzed the modulation of ERK1/2 and AKT cascades by means of Western blotting. As showed in Fig. 4, *R*. *graveolens* a.e. increased ERK1/2 phosphorylation in all the glioma cell lines analysed as well as in non-differentiated A1 cells. In particular, as compared to control conditions, ERK1/2 phosphorylation was increased by 4-folds in C6 cells, as early as 5 min after the addition of the *R*. *graveolens* extract. The activation of ERK1/2 lasted up to 30 minutes with a further 1,5 fold increase 60 min after the treatment (Fig. 4A). In U138 cells, *R*. *graveolens* induced a significant increase in ERK1/2 phosphorylation 5 and 10 minutes after treatment with a return to control levels 60 minutes after treatment (Fig. 4D). Similar results were obtained in A1 cells (Fig. 4C) and in U87MG cells (Fig. 4D) in which after a rapid activation of ERK1/2, longer treatments resulted in a partial reduction of the effect. These results suggest that the antiproliferative effects of *R*. *graveolens* could be related to activation of the ERK1/2 pathway. Being this result rather unexpected, we verified this relationship analyzing cell proliferation in the presence of the selective MEK inhibitor PD98059. MTT assay performed on U87MG, U138, C6 and A1 cells treated or not with 1mg/ml *R*. *graveolens* a.e. for 48h in the presence of PD98059 (10μM), revealed that the inhibition of ERK1/2 pathway rescued cell viability impairment induced by *R*. *graveolens* a.e. treatment (Fig. 4E-H).

**Fig 5:**
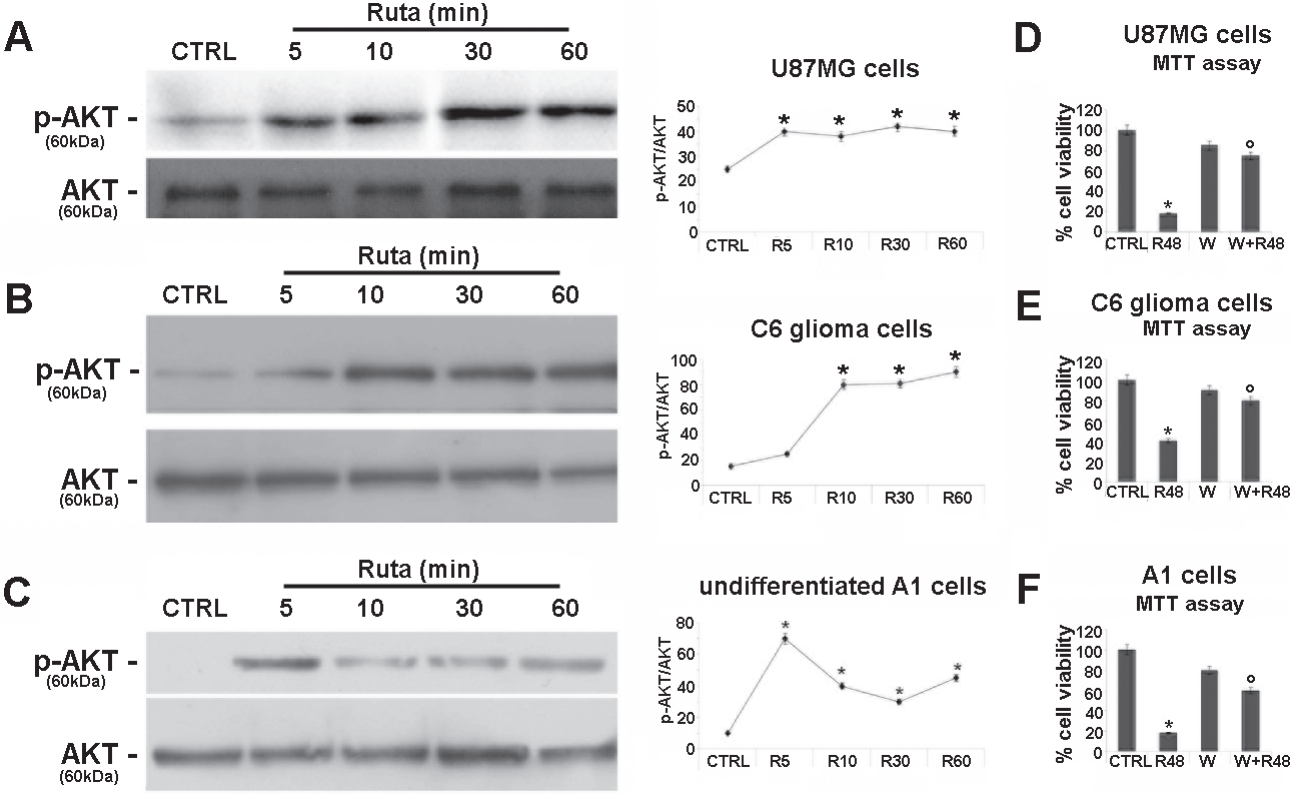
*R*. *graveolens* a.e. is able to induce AKT phosphorylation in glioma and proliferating neural cells. (A) Western blotting detection of p-AKT and AKT proteins in U87MG (A), in C6 glioma cells (B) and in A1 cells (C) treated with 1mg/ml *R*. *graveolens* a.e. for 5, 10, 30 and 60 minutes. A specific band is observed at 60 kDa. Each blot is representative of three separate experiments. The graphs show the relative quantitation of p-AKT and AKT in the different cell lines. Data are expressed as ratios of p-AKT/AKT. Asterisks represent p<0.05 *vs* controls. (D-E) MTT assay in U87MG cells (D), C6 glioma cells (E) and A1 cells (F) treated for 48 hours with 1mg/ml *R*. *graveolens* a.e. (R48) or with 1μM wortmannin (W), or in combination (W+R48); *p<0.01 *vs* control conditions.°p<0,05 *vs* R48.

We also evaluated the role of AKT in the *R*. *graveolens* effects. AKT phosphorylation as index of kinase activation, was assessed in U87MG, C6 and proliferating A1 mes c-myc cells, by Western blot analysis. As shown in Fig. 4, the treatment with *R*. *graveolens* significantly increased also AKT activation, showing a sustained phosphorylation for all the length of the treatment (up to 60 minutes). However, while in U87MG and C6 glioma cells (Fig. 5A, B) AKT phosphorylation status remained stable at the peak levels, in A1 mes c-myc cells (Fig. 4C), after a rapid (5 minutes) 2-fold increase, AKT phosphorylation partially declined, although remaining at a significantly higher levels as compared to control conditions, up to 60 minutes.

To confirm the involvement of PI3K/AKT pathway in the antiproliferative effect of *R*. *graveolens* a.e., cell proliferation was assessed in the presence of the selective PI3K inhibitor wortmannin. MTT assay performed on U87MG, C6 and A1 cells treated or not with 1mg/ml *R*. *graveolens* a.e. for 48h in the presence of wortmannin (1μM). These experiments revealed that also the inhibition of *R*. *graveolens*-activated AKT rescued cell viability impairment as compared to cells treated with *R*. *graveolens* a.e. alone (Fig. 5 D-F). The preincubation with the combination of the two inhibitors (PD98059 and wortmannin) did not induce a higher protective effect on the cells viability (data not shown), suggesting that the two pathways do not cooperate.

### *R*. *graveolens* aqueous extract induces apoptosis in proliferating/undifferentiated A1 mes-c-myc cells.

**Fig 6:**
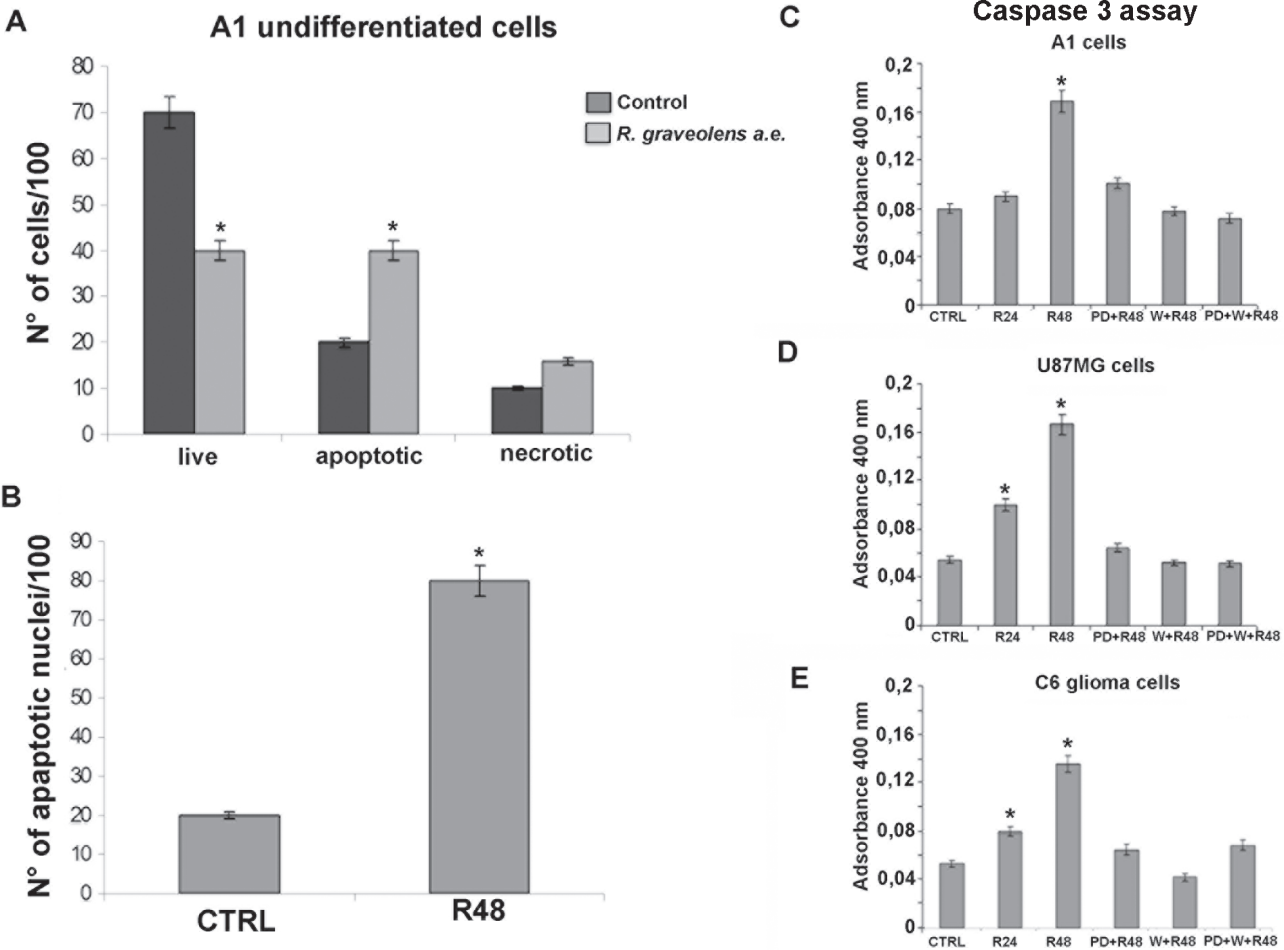
*R*. *graveolens* a.e. induces apoptosis in A1 cells. (A) Cell cycle was analyzed by means of Tali image-based cytometry on proliferating A1 cells in control conditions (dark grey) and 48h after 1mg/ml *R*. *graveolens* a.e. treatment (light grey) *p<0.01 *vs* controls (B) Number of apoptotic nuclei/100 cells treated (R48) or not (CTRL) with 1 mg/ml *R*. *graveolens* a.e. for 48 hours. *p<0.01 *vs* controls. (C-E) Caspase 3 activity expressed as absorbance at 400 nm in A1 cells (C), U87MG cells (D) and C6 cells (E) treated with vehicle (CTRL), 1mg/ml *R*. *graveolens* a.e. for 24 (R24) or 48 (R48) hours, 10μM PD98059 in combination with ml *R*. *graveolens* a.e. for 48 hours (PD+R48), 1μM wortmannin in combination with ml *R*. *graveolens* a.e. for 48 hours (W+R48) or the combination of the two inhibitors (PD+W+R48) for 48 hours. *p<0.01 *vs* control conditions.

In order to elucidate the type of cell death induced by *R*. *graveolens* a.e., the analysis of annexin V binding and propidium iodide (PI) nuclear staining was used to discriminate between apoptotic and necrotic cell death, using Tali image-based cytometry. As shown in Fig. 6A, *R*. *graveolens* a.e. (1mg/ml), administered for 48 h, significantly increased the number of apoptotic A1 cells as compared to vehicle-treated cells. Noteworthy, no changes in the number of necrotic cells was observed in treated and control cells (Fig. 6A). These data were confirmed evaluating nuclear morphology after DAPI staining, in A1 cells before and after treatment with *R*. *graveolens* a.e. (1mg/ml) for 48 hours. As shown in Fig. 6B, the number of apoptotic nuclei (i.e. showing condensed and/or fragmented morphology) was significantly higher (+400%) in treated cells as compared to control conditions. Altogether, these data indicated that *R*. *graveolens* a.e. was able to induce cell death in proliferating A1 cells activating the apoptotic program. To corroborate this finding we measured caspase 3 activity in A1, U87MG and C6 cells. As shown in Fig. 6 (C-E), *R*. *graveolens* a.e. treatment increased caspase 3 activity of about 100% over control cells in A1 mes c-myc cells and caused a 4-fold induction in U87MG and C6 cells. Moreover, preincubation with PD98059, wortmannin, or both inhibitors completely abolished this effect in both A1 and glioma cells (Fig. 6 C-E). The treatment with either inhibitor alone did not modulate caspase 3 activity as compared to untreated cells (data not shown).

### Rutin, a major component of *R*. *graveolens* extract, does not affect proliferating A1 cells viability.

**Fig 7:**
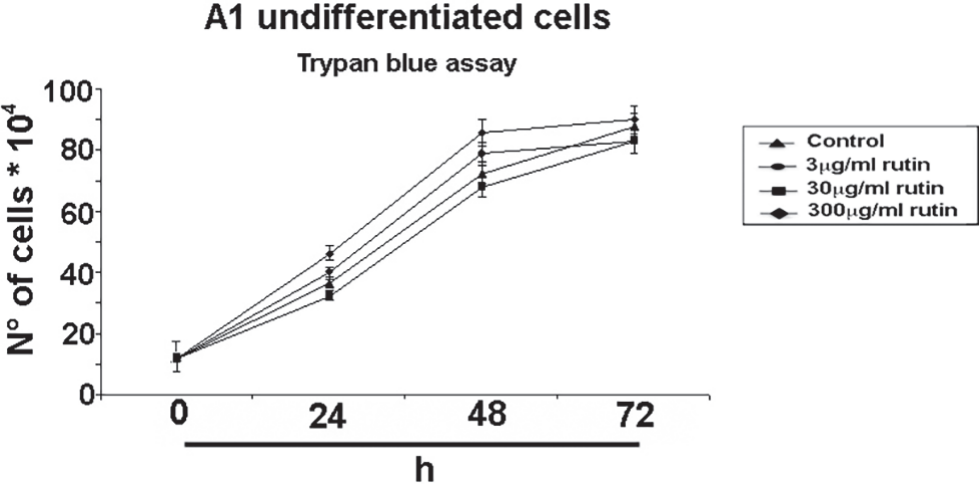
Rutin does not influence proliferating A1 cells viability. Trypan blue exclusion test on proliferating A1 cells treated with or without (control) increasing concentrations of rutin (3μg/ml, 30μg/ml and 300μg/ml) for 24, 48 and 72h.

Rutin is a major component of *R*. *graveolens* extract [55]. Thus, we tested the effect of rutin on A1 undifferentiated/proliferating cells. MTT (data not shown) and Trypan blue (Fig. 7) assays demonstrate that rutin treatment (3μg/ml, 30μg/ml and 300μg/ml for 24, 48 and 72h) does not affect proliferating A1 cell viability, and suggest that rutin by itself is not responsible of the cell death induced by *R*. *graveolens* a.e.

## Discussion and Conclusions

A water extract from *R*. *graveolens* induces caspase 3-dependent apoptotic cell death of human and rat glioblastoma cells and of a neural progenitor cell line (A1 cells) derived from embryonic mouse CNS. Interestingly, upon differentiation and cell cycle exit, A1 cells become insensitive to the noxious effects of *R*. *graveolens* extract. GBM aggressive behaviour and poor prognosis is partly due to its peculiar ability to massively invade surrounding brain tissues preventing a complete surgical excision. The property of *R*. *graveolens* extract to discriminate between proliferating (i.e. tumor cells) and non-proliferating neural cells, as shown in this study, has a potential to be exploited as therapeutic tool in GBM. Further experiments, especially exploring at a molecular level these effects, are, however, required to definitely ascertain the absence of noxious effects on fully differentiated, non-proliferating neurons. It is worth noting that it was previously shown that *R*. *graveolens* extract or some of its components were able to exert anti tumor activity *in vivo* and *in vitro* [56, 57]. In particular, R. graveolens extract in combination with calcium phosphate was shown to induce cell death of glioma cells but proliferation of peripheral blood limphocytes [56] Although this study provided data showing that rue extract exerts both *in vitro* and *in vivo* therapeutic effects on brain tumors, it has been basically ignored in the literature. The reasons for that may depend upon different matters. Indeed, this study does not provide clues on the molecular mechanism(s) underlying the observed therapeutic effects. Moreover, rue extract was administered as a omeopathic solution in combination with CaPO4 and no explanation was given for that protocol. In addition, in literature there are contrasting results on the genotoxic and clastogenic effects of R. graveolens extract on healthy and tumor cells [58]. Thus, our study may provide a new spin on the use of R. graveolens extracts as a therapeutic tool. To this regard, it is worth noting that the concentrations of rue extract used in our experiments are significantly lesser than those tested by Preethi [58], and, in addition, we indicate a signal transduction pathway involved in the effects. Finally, it is of note that differently from the studies of Pathak [56] and Preethi [58] who tested the pharmacological effect of the alcoholic extract of R. graveolens, our study reported the antiproliferative effect of the water extract of the plant.

Drugs used to treat GBM, including temozolomide [59] and cisplatin [60], two alkylating agents that target the DNA of both cancer and normal cells and are currently used in first- and second-line treatment, respectively, cause cognitive impairment such as loss of memory and learning, which are even more severe in the case of childhood brain. Although the underlying cellular and molecular mechanisms are largely unknown, these side-effects, often referred to as “chemo-brain”, are likely due to normal brain cells damages [61]. Importantly, in our model these drugs, differently form *R*. *graveolens* extract, were not able to discriminate among glioma or proliferating A1 cells and differentiated A1 cells causing a significant cell death in all the cell populations analysed. Moreover, tumours treated with temozolomide or cisplatin develop chemo-resistance largely due to high methyl-guanine methyl transferase (MGMT) levels and to inactivation of mismatch repair enzymes MLH1 and MSH29 and over-expression of multidrug resistance proteins, respectively [61]. Thus, it is important to explore new, more efficacious and less toxic therapeutic approaches. Terrestrial and marine life can generate a number of compounds, termed secondary metabolites, in response to intense ecological competition in order to survive in their environment. Most of these compounds are endowed with biological properties, including anti-cancer and/or chemo-preventive effects [36, 37]. Preclinical and clinical studies have shown that water or alcoholic natural extracts, as a whole and/or molecules isolated from such extracts, can exert a number of different effects useful to fight tumors. In particular, natural compounds can exert anti-cancer activity by means of different mechanisms: i) directly killing transformed cells; ii) acting synergistically with classical chemotherapy by improving its efficacy and, thus, allowing a lower dose of drug to be effective; iii) reverting the drug-dependent resistance; iv) providing antioxidant molecules; and iv) activating and/or enhancing endogenous anti-cancer defences, including immune system. A specific goal of anti-cancer research is to find natural products endowed with toxicity against cancer cells but harmless to healthy cells. Although many natural products are promising therapeutic tools since in preclinical studies they were shown to kill transformed cells, nevertheless few data are present about their effects on normal tissues, in particular as far as CNS is concerned [62].

With regard to selectivity, in a previous collaborative study, together with marine eco-physiologists and chemists, we reported that a compound extracted from microalgae exerts very different effects according to the differentiation status of the cells [63]. In particular, 2–4, trans-trans decadienal, an aldehyde presents in the microalgae diatomee, produces its noxious effects on copepods, tiny crustaceans their natural predators, in a manner that is dependent upon cell differentiation. Thus, diatomee-derived aldehydes are harmless towards adult copepods whereas when used to feed mothers, they are teratogen to larvae, causing anatomic malformations and death of eggs. We showed that this differentiation-dependent mechanism is maintained along the phylogenies. In fact decadienal causes apoptotic cell death in the undifferentiated, proliferating mammalian neural cell line A1, generated from embryonic mouse mesencephalon, whereas, upon differentiation and cell cycle exit, the same cells become unresponsive to the noxious effects of the same compound. It seems that 2,4, trans-trans decadienal and *R*. *graveolens* share the same selective effects on proliferating/undifferentiated and non-proliferating differentiated neural cells, at least in the A1 cellular model.

Rutin, the major component of *R*. *graveolens* water extract that we administered on cells, was not able, even at high concentrations, to affect the viability of A1 cells grown under proliferating, undifferentiated cells, thus indicating that either rutin is necessary but not sufficient to elicit cell death or even that other compounds present in minor amounts in *R*. *graveolens* a.e. are responsible for most of the observed biological effects. Further experiments will assess the involvement of rutin and/or of other compounds within *R*. *graveolens* water extract in determining the biological effects observed.

Interestingly, as far as *R*. *graveolens* antiproliferative intracellular signalling, we show the *R*. *graveolens* extract stimulates the phosphorylation/activation of both ERK1/2 and AKT. A number of natural compounds are able to interfere with ERK1/2 and AKT activation. In turn, in many instances, perturbation of ERK1/2 and AKT signalling is responsible for anti-cancer effects. ERK1/2 and AKT signalling have been classically related to cell proliferation and survival. Indeed many mutated oncogenes cause the constitutive activation of these pathways and contribute to cell transformation [64–67]. In fact, selective ERK1/2 and AKT inhibitors or receptor-mediated reduction of the activity of these kinases have been shown to halt uncontrolled cell proliferation or to kill transformed cells [68–74]. Nevertheless, the biological effects of ERK1/2 and AKT are cell context- and/or stimulation mode-dependent. For instance, as far as ERK1/2 is concerned, its activation can elicit, on the one hand, either proliferation or differentiation and cell cycle exit, and on the other, either cell survival or cell death, according to the intensity and/or timing of stimulation [75, 76]. We found that *R*. *graveolens*-induced cell death decreases, in both glioma and A1 cells, upon blocking, by means of selective inhibitors, ERK1/2 or AKT, thus showing that, in our experimental conditions, ERK1/2 and AKT, at least partly, mediate the toxic effects. Similar results were obtained analyzing caspase 3 activity induced by *R*. *graveolens*. Indeed, caspase 3 was abolished upon inhibition of ERK1/2 and/or AKT activity by PD98059 or wortmannin. As a possible mechanism by which increased ERK activity may result in antiproliferative effects, it was reported, in a different cell context (i.e. CHO cells), that the antiproliferative activity of somatostatin was related to ERK1/2-dependent induction of the cell cycle inhibitor p21^cip1^ [77]. Further experiments are required to identify the molecules involved also in the signalling cascade originated by *R*. *graveolens*. However, a similar paradoxical activation of both ERK1/2 and AKT was reported in glioma cell lines to mediate the antiproliferative activity of adiponectin [78], confirming that in glioma cells over-activation of these kinase cascades is responsible of antiproliferative effects. Moreover, in A549 lung carcinoma cells, quercetin, a metabolite in the degradation pathway of rutin, was shown to induce activation of ERK and, as a consequence, of caspase-3 and apoptosis. In turn, activation of MEK-ERK was required for quercetin-induced apoptosis [79].

Finally, although the precise mechanisms of the anti-proliferative action of rue extract are unknown, there are evidence suggesting that the action of flavonoids might be mediated by their interaction with aryl hydrocarbon receptor or estrogen binding sites, which have been shown to be occupied by flavonoid-like molecule with growth inhibitory properties [80–82].

In conclusion, our data show that *R*. *graveolens* a.e. is endowed with potent antitumoral activity in human glioma cells and in undifferentiated cells originated from mouse embryonic brain, whereas it results harmless towards the same differentiated, non-proliferating cell line. Thus, it represents a potential new therapeutic tool for brain cancer therapy and also for investigating the molecular events underlying the toxicity of neural cells to exogenous compounds.

## Supporting Information

S1 Fig *R*. *graveolens* extract induces cell death of C6 and of human U138 glioma cells. MTT assay on proliferating C6 glioma cells (A) and U138 human glioma cells (B) treated with vehicle (♦) or with 1mg/ml *R*. *graveolens* a.e. (■), *p<0,01 vs control conditions.
(TIF)
