# Supplementary figures and images for: Ruta graveolens L. Induces Death of Glioblastoma Cells and Neural Progenitors, but Not of Neurons, via ERK 1/2 and AKT Activation
Source: PLoS One. 2015 Mar 18;10(3):e0118864. doi: 10.1371/journal.pone.0118864 (PMC4364962; doi:10.1371/journal.pone.0118864)

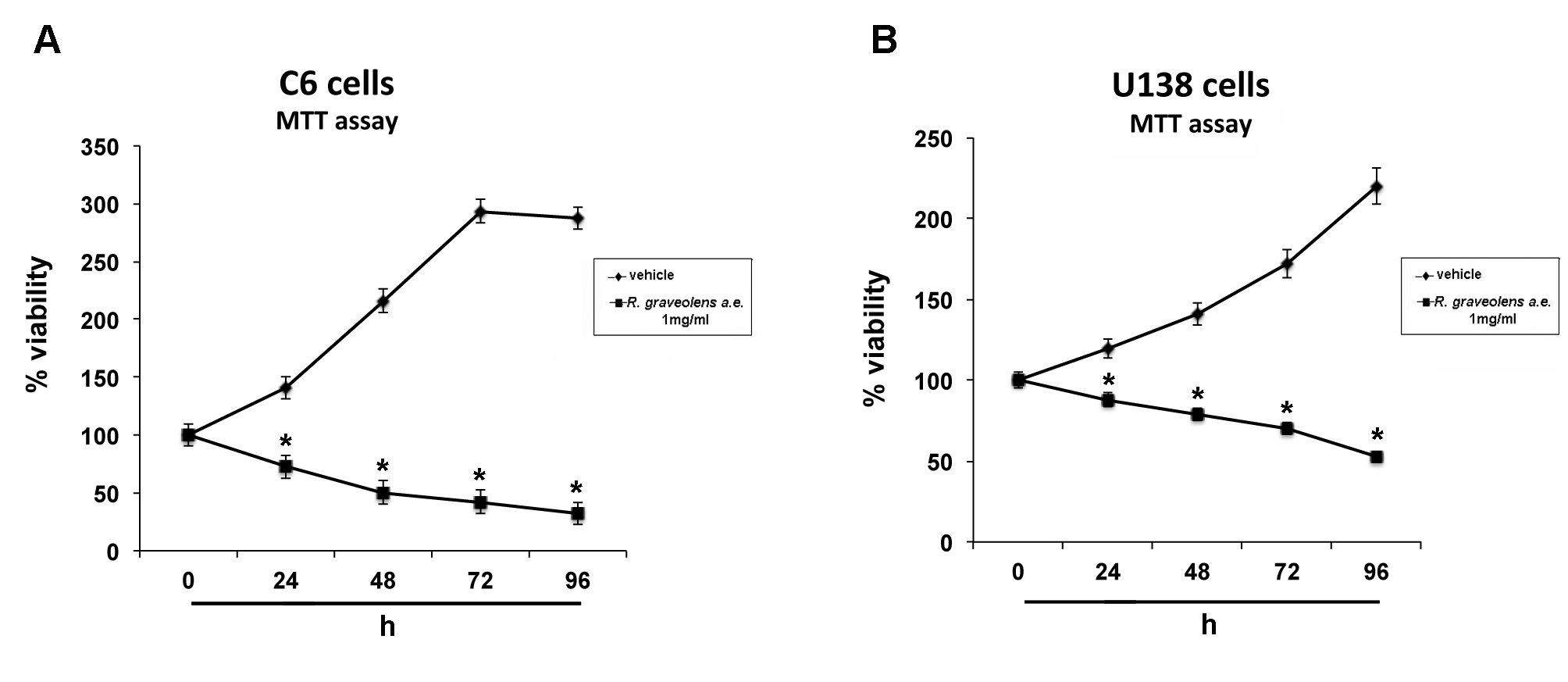

Supplement: S1 Fig — MTT assay on proliferating C6 glioma cells (A) and U138 human glioma cells (B) treated with vehicle (♦) or with 1mg/ml R. graveolens a.e. (■), *p<0,01 vs control conditions. (TIF) [file pone.0118864.s001.tif]
